# The association between bariatric surgery and extensive portal vein thrombosis: A case report

**DOI:** 10.1016/j.ijscr.2024.110276

**Published:** 2024-09-10

**Authors:** Muzi Meng, Jigyasha Pradhan, Ajit Singh

**Affiliations:** a General Surgery, BronxCare Health System, Bronx, NY, USA; b School of Medicine, American University of the Caribbean, Cupecoy, Sint Maarten (Dutch Part)

**Keywords:** Portal vein thrombosis, Venous thrombosis, Bariatric surgery, Anticoagulation

## Abstract

**Introduction and importance:**

As the obesity rate continues to rise, portal vein thrombosis (PVT) has emerged as a more frequent complication following bariatric surgery, with an incidence reported at approximately 0.4 % according to recent meta-analyses. PVT, characterized by the development of a thrombus within the portal vein, can be life-threatening due to its subtle and often nonspecific symptoms, complicating timely diagnosis and treatment.

**Case presentation:**

In this case report, we present a 45-year-old female patient with a history of morbid obesity who underwent robotic-assisted laparoscopic sleeve gastrectomy and hiatal hernia repair. On postoperative day 16, she developed symptoms of severe abdominal pain and intolerance to oral intake, suggesting the presence of portal vein thrombosis. Laboratory findings showed significantly elevated D-dimer levels, and contrast-enhanced CT imaging confirmed an extensive thrombus within the portal vein. The patient was promptly admitted to the critical care unit, where she was managed conservatively with therapeutic anticoagulation, including subcutaneous heparin preoperatively and postoperatively, and discharged with a prescription for apixaban.

**Clinical discussion:**

Early diagnosis of PVT in the post-bariatric population is critical, as it allows for timely intervention with evidence-based therapeutic options such as anticoagulation, thereby improving both short- and long-term patient outcomes. This case not only underscores the importance of heightened vigilance for PVT in patients presenting with nonspecific abdominal symptoms after bariatric surgery but also highlights the potential risk factors unique to this patient, such as prolonged operative time and underlying comorbidities, which may have contributed to the thrombotic event. A multidisciplinary approach, involving both medical and surgical teams, is essential for optimal management of such complex cases.

**Conclusion:**

This case underscores the critical importance of early recognition and prompt management of portal vein thrombosis in post-bariatric surgery patients. By emphasizing the role of thorough perioperative DVT prophylaxis, including the use of heparin and sequential compression devices, this report not only aims to improve patient outcomes but also contributes to the growing body of knowledge on the prevention and treatment of PVT in the bariatric population. These insights may serve as a valuable framework for managing similar clinical scenarios in the future.

## Highlights

• Bariatric surgery is a preferred treatment option for obese patients.
• Obese patients are at risk of developing portal vein thrombosis formation.
• Physicians need to be highly suspicious of any symptom of portal vein thrombosis developed by patients.
• The wait-and-see approach is preferable for treating patients with portal vein thrombosis.

## 1 Introduction

Portal vein thrombosis (PVT) is a significant and potentially life-threatening condition characterized by the formation of a blood clot within the portal vein, which can impede blood flow to the liver [1]. Boccatonda et al. reported that the portal vein is the most common site for thrombosis formation compared to other veins [1]. Bariatric surgery, a common and effective treatment for morbid obesity, has been associated with multiple complications, including PVT [2]. The incidence of PVT after laparoscopic surgery, including bariatric procedures, has been reported at around 0.4 %, largely due to the pneumoperitoneum causing sluggish blood flow in the portal vein [3]. This increase highlights the need for heightened clinical awareness and effective management strategies for PVT in this patient population. In this case report, we present a specific patient case to explore the association between bariatric surgery and PVT. We discuss potential risk factors, including surgical techniques, comorbidities, and duration of surgery, which may have contributed to the development of PVT in this case. By reviewing these factors, this report aims to provide insights into the effective management strategies for patients with PVT following bariatric surgery. This work has been reported in line with the SCARE criteria [4].

## 2 Case presentation

A 44-year-old female with a past medical history of morbid obesity, mild obstructive sleep apnea, and hypothyroidism, 16 days post robotic-assisted laparoscopic sleeve gastrectomy and hiatal hernia repair presented to the emergency department (ED) because of acute upper abdominal pain and middle back pain. Patient reported the pain was located in epigastric area radiating to the back, was getting A 44-year-old female with a past medical history of morbid obesity, mild obstructive sleep apnea, and hypothyroidism, presented to the emergency department (ED) 16 days post robotic-assisted laparoscopic sleeve gastrectomy and hiatal hernia repair due to acute upper abdominal pain and middle back pain. The patient reported that the pain was located in the epigastric area, radiating to the back, and had been worsening over the past five days. She denied fever, chills, shortness of breath, chest pain, acid reflux, nausea, vomiting, or dysuria. She had been unable to tolerate any solid food, but could tolerate liquids until the day prior to admission. Upon presentation to the ED, the patient was afebrile, normotensive, but tachycardic, with a heart rate of 120 beats per minute.

Laboratory results showed an elevated white blood cell (WBC) count of 11,600/μl, lactic acid level of 0.8 mmol/L, and a plasma D-Dimer level of 3994 ng/mL, with a partial thromboplastin time (PPT) of 26.2 s, prothrombin time (PT) of 17.8 s, and an international normalized ratio (INR) of 1.46.

**Fig. 1:**
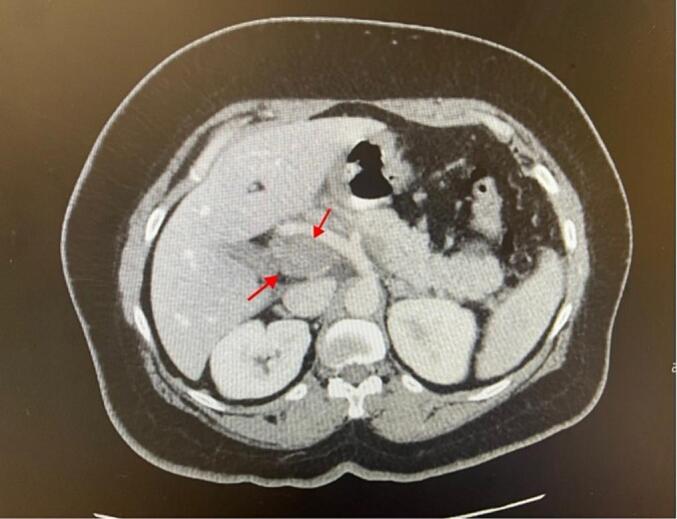
CT Abdomen and Pelvis with Contrast showed the extensive portal vein thrombosis (red arrows). (For interpretation of the references to colour in this figure legend, the reader is referred to the web version of this article.)

A contrast-enhanced computed tomography (CT) scan revealed no free air, no evidence of a leak or extravasation, but showed extensive venous thrombosis involving the main portal vein as well as the right and left intrahepatic portal veins, the splenic vein, and the superior mesenteric vein [Fig. 1]. The CT scan also confirmed that the patient did not have any focal lesions in her spleen, no peripancreatic fat stranding, no abdominal aortic aneurysm, and no retroperitoneal hematoma. Additionally, there was no bowel dilatation, bowel thickening, or intestinal obstruction.

**Fig. 2:**
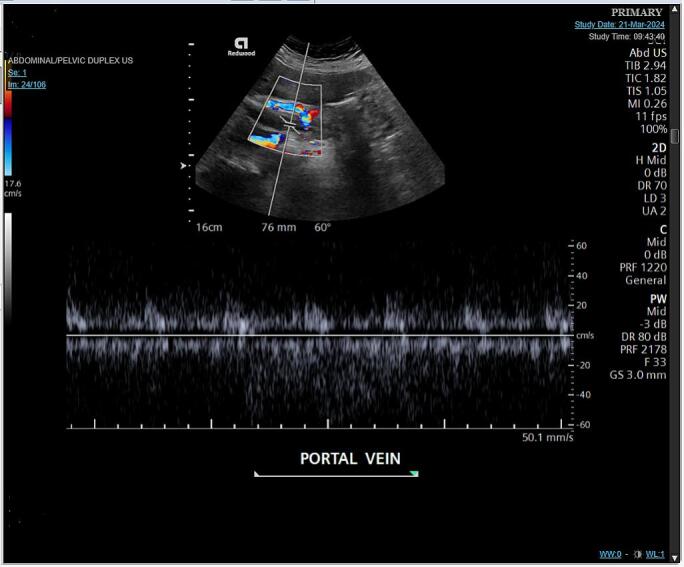
Ultrasound abdomen and pelvis Doppler venous duplex showed thrombosed portal vein.

The patient was admitted to the critical care unit (CCU) and kept NPO (nothing by mouth). She was started on therapeutic enoxaparin 90 mg twice daily. Hematology and vascular surgery consultations were obtained, and an abdominal venous duplex ultrasound confirmed the thrombosed portal vein [Fig. 2]. A lower extremity venous duplex ultrasound was also performed, which was negative for deep venous thrombosis (DVT). A repeat CT angiogram showed a diminished thrombus in the portal vein, with no change in the superior mesenteric and splenic veins.

On the fourth day of hospitalization, the patient was started on bariatric clears, defined as 90 cm^3^–120 cm^3^ of clear liquids per hour (e.g., water, clear broth, and clear juices). She was discharged on the sixth day with a prescription for apixaban (Eliquis) 5 mg twice daily for three months. The patient followed up with the outpatient clinic, where it was planned that a CT angiogram would be repeated toward the end of the three-month course of apixaban, and the continuation of anticoagulation therapy would be determined based on the findings.

## 3 Discussion

Portal vein thrombosis (PVT) is the formation of a blood clot within the portal vein, which is the most common site of venous thrombosis compared to other veins such as the splenic, mesenteric, or hepatic vein [1,5]. This condition can arise from various pathological conditions, including chronic liver diseases, systemic inflammatory diseases, and neoplasms. Anatomically, the portal vein forms behind the pancreas, ranges between 5.5 and 8 cm in length, and courses toward the porta hepatis before dividing into the right and left main branches [6]. Several risk factors that may trigger portal vein thrombosis include cancer, hepatic cirrhosis, and various local factors such as idiopathic origins, cholangitis, abdominal sepsis, surgical and non-surgical trauma, iatrogenic factors, malignancy, pancreatitis, perinatal omphalitis, estrogen therapy, and portal hypertension [6].

Prakash et al. describe that PVT may develop as a consequence of a hypercoagulable and hyperinflammatory state, exacerbated by deficiencies in anticoagulants such as vitamin C and protein S [5]. The complexity of PVT is further highlighted by its resemblance to intimal fibrosis, the formation of fibrous connective tissue in the innermost layer of arteries, making it more intricate than systemic venous thrombi [5]. Although the condition is rare, it can be life-threatening due to its typically limited symptoms, complicating the diagnosis. Kowalski et al. classified the risk factors for PVT into two groups: systemic (e.g., coagulation disorders, obesity, and pregnancy) and local (e.g., trauma, infection, and local inflammation) [7]. Obesity and coagulation disorders, such as thrombophilia and hemophilia, significantly increase the risk of developing PVT [8]. Additionally, surgical procedures, particularly bariatric surgeries, may precipitate PVT, especially in patients with pre-existing thrombosis risk factors.

Laparoscopic surgeries, commonly used in bariatric procedures, increase intra-abdominal pressure, which leads to a decrease in blood flow in the portal vein, creating an ideal condition for thrombosis to form [7]. Godoroja-Diarto et al. highlighted that despite advancements in laparoscopic techniques, venous thromboembolic complications, including PVT, have not been effectively minimized, with the incidence of hereditary thrombophilia in bariatric patients ranging from 2 % to 52 % [8]. Due to the life-threatening potential of extensive portal vein thrombosis in these patients, it is crucial for physicians to maintain a high level of suspicion for prompt diagnosis and treatment [8].

Furthermore, the incidence of PVT after laparoscopic surgery, particularly in bariatric procedures, has been reported at approximately 0.4 %, as noted in a recent meta-analysis by Luo MS et al. [9]. The pneumoperitoneum required during laparoscopic surgery, which causes sluggish blood flow in the portal vein, is a contributing factor. Given the rising prevalence of bariatric surgery among patients with morbid obesity, understanding the relationship between surgical interventions and PVT is crucial.

Bariatric surgery, such as duodenal switch or Roux-en-Y gastric bypass, has become a popular standalone treatment for obesity [8]. However, these procedures, particularly when performed laparoscopically, may lead to severe complications, including PVT. Complications associated with these surgeries include increased intra-abdominal pressure, carbon dioxide pneumoperitoneum, vomiting, nausea, diarrhea, and constipation [9,10]. Moreover, obesity itself is a hypercoagulable state associated with an increased risk of thrombotic events [10], suggesting that obese patients undergoing bariatric surgery are at a heightened risk of developing PVT.

Given the high risk of thrombotic complications in this population, DVT prophylaxis, including the use of subcutaneous heparin and sequential compression devices (SCDs), is essential. In this case, preoperative and postoperative DVT measures, including subcutaneous heparin 5000 units and the use of SCDs, were implemented. The patient was also encouraged to ambulate on the day of surgery. Despite these measures, the patient developed extensive PVT, which underscores the need for vigilant postoperative monitoring and early intervention.

The treatment in this case was successful with conservative management using anticoagulation therapy. The patient was managed with enoxaparin followed by apixaban for three months. The decision to continue anticoagulation therapy beyond this period was based on follow-up imaging findings. In cases where conservative management is unsuccessful, surgical interventions may be required. This case highlights the importance of early recognition and a multidisciplinary approach in managing PVT following bariatric surgery.

Al-Sawabkeh et al. noted that patients undergoing bariatric surgery are at an increased risk of venous thromboembolism (VTE) due to the inflammatory and hypercoagulable conditions associated with obesity [11]. Sympathetic vasoconstriction, which leads to the release of vasopressors, can reduce venous blood flow, thereby increasing the risk of thrombosis, including extensive portal vein thrombosis (PVT). Other contributing factors to post-bariatric surgery PVT include metabolic syndrome and the inherent thromboembolic risks associated with obesity [11]. Wilkinson et al. found that PVT typically occurs between 8 and 19 days postoperatively in women aged 36–47 years, with the likelihood of developing the condition depending on each patient's specific bleeding risk factors [12].

Carrano et al. confirmed that portomesenteric venous thrombosis is a common complication following bariatric surgery, particularly in patients with underlying conditions such as myeloproliferative disorders, anticoagulant protein deficiencies, prothrombotic gene mutations, and portal hypertension [3]. PVT after bariatric surgery often presents mildly but can become more severe over time, underscoring the importance of early identification and treatment to minimize its impact [3]. The global increase in obesity has led to a rise in bariatric surgeries, and consequently, a rise in the prevalence of post-bariatric surgery PVT [13]. Tan et al. identified postoperative factors such as post-discharge dehydration and hypovolemia, which contribute to the formation of portal vein thrombosis [13].

Of all post-bariatric surgery PVT cases, approximately 80 % occur within the first 30 days after surgery, with the average onset being between 15 and 20 days [14]. Palomares et al. emphasized that diagnosing PVT requires a high level of suspicion from healthcare professionals, as the condition often presents with nonspecific symptoms such as abdominal pain, bloating, vomiting, and dehydration [14]. Barros et al. added that abdominal pain indicative of PVT may manifest days, weeks, or even months after bariatric surgery, although many cases remain asymptomatic [15]. Their study also confirmed that the portal vein is the most frequently affected site for abnormalities emerging from bariatric surgery.

Anticoagulant therapy, specifically continuous intravenous heparin infusion, is a common treatment for PVT, particularly during the initial phase when there is no active bleeding [6]. This therapy is typically administered for at least six months, depending on the underlying cause of the thrombosis. Interventional techniques, such as mechanical thrombectomy, are also being explored for their efficacy in treating PVT. Mechanical thrombectomy can dissolve the thrombus, preparing patients for local pharmacologic thrombolysis, making it a preferred option over more invasive procedures due to its effectiveness and lower risk profile [6].

Xie et al. emphasized that several factors must be considered before determining the most appropriate treatment for patients with PVT, including the extent of the thrombosis, clinical manifestations, complications from portal hypertension, and the risk of bleeding [16]. Acute thrombosis may require the timely administration of antithrombotic agents to recanalize the portal vein and prevent thrombus extension [16]. Surgery should be considered a last resort, reserved for cases where antithrombotic therapy fails, and intestinal ischemia or necrosis develops [16]. Anticoagulation therapy should also be administered after any gastroesophageal variceal bleeding if present. However, in some cases, a wait-and-see approach may be ideal, as mural thrombosis can resolve spontaneously without the need for anticoagulation therapy [16].

Costache et al. noted that anticoagulants are the most effective therapeutic option for restoring vascular flow and preventing thrombus formation and enlargement [17]. The choice of anticoagulant should be based on the location of the obstruction, the extent of the thrombosis, and the duration of the thrombotic episode. Low molecular weight heparin (LMWH) is often the most viable option due to its ability to reduce bacterial inflammation markers [17]. For non-cirrhotic, non-malignant PVT cases, thrombolytic therapy has shown high success rates, especially in patients with specific symptoms such as extensive bleeding. However, thrombectomy is generally not recommended due to its associated risks, although thrombus aspiration during transjugular intrahepatic portosystemic shunt (TIPS) has shown success in clinical trials [17].

## 4 Conclusion

Extensive portal vein thrombosis (PVT) is a rare but serious complication that can arise following bariatric surgery. The potential for complete blockage of the portal venous system underscores the severity of this condition. Given the nonspecific symptoms associated with PVT, such as abdominal pain, bloating, and vomiting, it is crucial for healthcare professionals to maintain a high index of suspicion, particularly in post-bariatric surgery patients. In the case presented, the patient fell within the demographic most vulnerable to post-bariatric surgery PVT, highlighting the need for vigilance in similar cases. The increasing prevalence of bariatric surgery as a treatment for morbid obesity likely contributes to the rise in PVT cases, making it essential for clinicians to recognize the signs early. Anticoagulation therapy remains the cornerstone of PVT management, though a tailored approach considering individual patient factors, such as comorbidities and the extent of the thrombosis, is vital. In some cases, a conservative, wait-and-see approach may be warranted to determine the most appropriate treatment strategy. This case report emphasizes the importance of a multidisciplinary approach, integrating medical, surgical, and imaging expertise to optimize patient outcomes in the management of PVT post-bariatric surgery.

## Consent

Written informed consent was obtained from the patient for publication and any accompanying images. A copy of the written consent is available for review by the Editor-in-Chief of this journal on request.

## Ethical approval

Ethics approval is not necessary since no novel treatment or detracting from standard treatment guidelines.

BronxCare Health System IRB.

## Funding

N/A.

## Author contribution

Muzi Meng-Writing-original draft preparation; writing-review & editing.

Jigyasha Pradhan-Writing-review & editing.

Ajit Singh-Attending Surgeon, Supervision.

## Guarantor

Ajit Singh.

## Research registration number

N/A.

## Conflict of interest statement

N/A.
